# Retraction: *Miscanthus* grass-derived carbon dots to selectively detect Fe^3+^ ions

**DOI:** 10.1039/d4ra90126f

**Published:** 2024-10-22

**Authors:** Maisyn Picard, Suman Thakur, Manjusri Misra, Amar K. Mohanty

**Affiliations:** a Bioproducts Discovery and Development Centre, Department of Plant Agriculture, University of Guelph Crop Science Building, 50 Stone Road East Guelph Canada mmisra@uoguelph.ca mohanty@uoguelph.ca; b School of Engineering, University of Guelph Thornbrough Building, 50 Stone Road East Guelph Canada

## Abstract

Retraction of ‘*Miscanthus* grass-derived carbon dots to selectively detect Fe^3+^ ions’ by Maisyn Picard *et al.*, *RSC Adv.*, 2019, **9**, 8628–8637, https://doi.org/10.1039/C8RA10051A.

The Royal Society of Chemistry, with the agreement of the authors, hereby wholly retracts this *RSC Advances* article due to concerns with the reliability of the data.

In Fig. 3 multiple sections of data are identical.

The four XRD patterns in Fig. 3a are comprised of the same individual components, but some sections have been flipped as mirror images to make the four traces appear different. For example, the data is identical in the region between 15 and 33° for all four traces, whereas in the region 37–46°, the data is identical in (i), (ii) and (iv), and is an identical mirror image in (iii). In the region 55–65°, the data is identical in (i), (iii) and (iv), but appears as an identical mirror image in (ii).

In Fig. 3b the Raman data is identical between 500 and 2200 cm^−1^ for (iv) and (ii) and between 500 and 1750 cm^−1^ for (iii) and (i). The data at 1750–2250 cm^−1^ in (iii) is an identical mirror image to the data in (i).

The second author, Suman Thakur, was responsible for all data in Fig. 3 and has admitted to data fabrication and image manipulation.

The authors did provide new data, which was assessed by an expert alongside the original paper who has indicated that the data no longer supports the conclusions of the paper.

Given the significance of these concerns, the findings presented in this paper are no longer reliable.

Signed: Suman Thakur, Amar K. Mohanty, Manjusri Misra and Maisyn Picard

Date: 9th October 2024

Retraction endorsed by Laura Fisher, Executive Editor, *RSC Advances*

